# Chronic thromboembolic pulmonary hypertension after acute pulmonary thromboembolism revealed by dynamic chest radiography

**DOI:** 10.1093/ehjci/jeac027

**Published:** 2022-02-07

**Authors:** Yuzo Yamasaki, Shohei Moriyama, Ryoma Tatsumoto, Kohtaro Abe, Kousei Ishigami

**Affiliations:** 1 Department of Clinical Radiology, Kyushu University, 3-1-1, Maidashi, Higashi-ku, Fukuoka 812-8582, Japan; 2 Department of Hematology, Oncology & Cardiovascular Medicine, Kyushu University, 3-1-1, Maidashi, Higashi-ku, Fukuoka 812-8582, Japan; 3 Department of Cardiology, Karatsu Red Cross Hospital, Karatsu, Japan; 4 Department of Cardiovascular Medicine, Graduate School of Medical Sciences, Kyushu University, 3-1-1, Maidashi, Higashi-ku, Fukuoka 812-8582, Japan

A 32-year-old woman on oral antipsychotics suddenly presented with cardiopulmonary arrest following acute dyspnoea and syncope. She recovered her spontaneous circulation after cardiopulmonary resuscitation in-ambulance during transit to a hospital. The patient was diagnosed with acute massive pulmonary thromboembolism (PTE) based on clinical presentation, elevated D-dimer level (9.5 μg/mL), and contrast-enhanced computed tomography findings (thrombi mainly located in the centres of the pulmonary arteries; *PanelsA* and *B*, arrows) in another hospital, and was referred to our hospital due to residual haemodynamic instability. Thrombolytic, catecholamine, and oxygen therapies were initiated immediately. Transthoracic echocardiography demonstrated right ventricular dilatation and left-sided deviation of the interventricular septum (parasternal short-axis view, Supplementary data online, *Video S1*). Dynamic chest radiography (DCR) was performed on Day 3 of hospitalization, revealing multiple triangular perfusion defects in bilateral lung fields (*PanelC*, Supplementary data online, *Video S2*). After gradual improvement, the patient was discharged on oral anticoagulants. The patient’s D-dimer level had normalized as of the time of discharge. However, she suffered from mild-effort dyspnoea even after 6 months; progression to chronic thromboembolic pulmonary hypertension (CTEPH) was suspected. Transthoracic echocardiography showed mild dilatation of right ventricle (parasternal short-axis view, Supplementary data online, *Video S3*). Repeat DCR demonstrated persistent large perfusion defects in bilateral lung fields (*PanelD*, Supplementary data online, *Video S4*), and it was very similar to the findings of the subsequently performed ventilation/perfusion (V/Q) scintigraphy (V/Q mismatch; *PanelsE* and *F*, arrows). Following re-admission, pulmonary hypertension was proven by invasive right heart catheterization (mean pulmonary arterial pressure 26 mmHg, pulmonary arterial wedge pressure 13 mmHg, Cardiac output 4.3 L/min, pulmonary vascular resistance 3.0 wu), and the diagnosis of CTEPH was confirmed.

**Figure:**
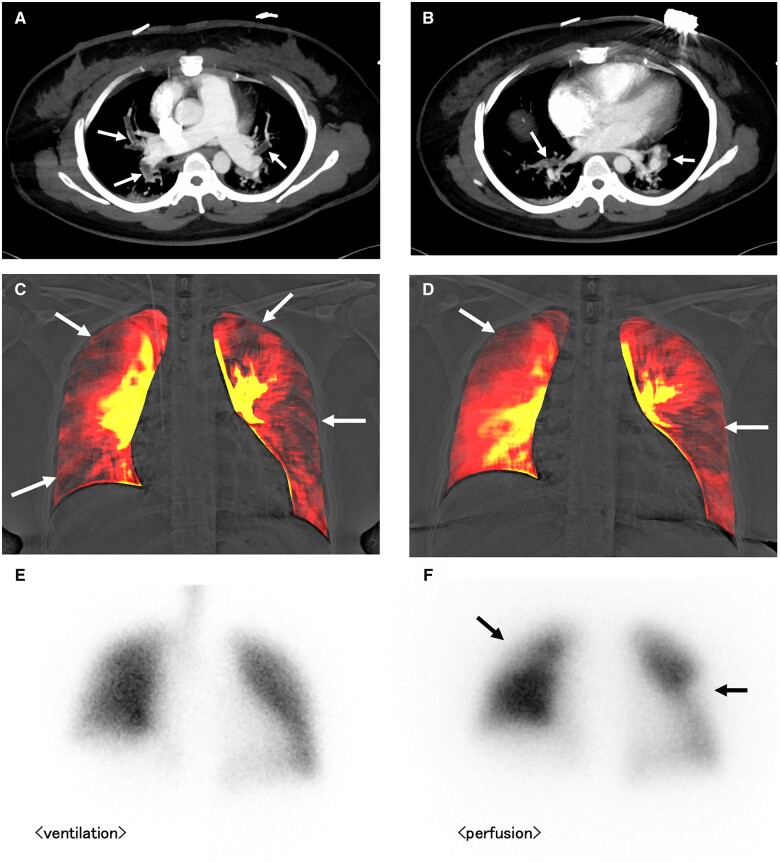


CTEPH is a relatively rare but serious complication after acute PTE. Although V/Q scintigraphy is recommended to detect residual perfusion abnormalities, it is not frequently used. DCR is a novel cineradiography technique using a flat-panel detector and a pulsed X-ray generator that shows pulmonary perfusion from temporal change in X-ray attenuation without any contrast media or radionuclide. The radiation exposure is much lower than that for V/Q and computed tomography scans. Moreover, it can be performed efficiently in outpatient settings. Conversely, the patient’s inability to keep still or to hold his or her breath for 7–10 s could lower the accuracy of the study. This is the first report that shows DCR-based detection of progression of acute PTE to CTEPH.

Supplementary data are available at *European Heart Journal - Cardiovascular Imaging* online.

## Supplementary Material

jeac027_Supplementary_Data

